# Ribociclib plus letrozole versus letrozole alone in patients with de novo HR+, HER2− advanced breast cancer in the randomized MONALEESA-2 trial

**DOI:** 10.1007/s10549-017-4518-8

**Published:** 2017-11-21

**Authors:** Joyce O’Shaughnessy, Katarina Petrakova, Gabe S. Sonke, Pierfranco Conte, Carlos L. Arteaga, David A. Cameron, Lowell L. Hart, Cristian Villanueva, Erik Jakobsen, Joseph T. Beck, Deborah Lindquist, Farida Souami, Shoubhik Mondal, Caroline Germa, Gabriel N. Hortobagyi

**Affiliations:** 1 Texas Oncology-Baylor Charles A. Sammons Cancer Center and US Oncology Network, 3410 Worth Street, Suite 400, Dallas, TX 75246 USA; 2 Masaryk Memorial Cancer Institute, Žlutý kopec 543/7, 656 53 Brno, Czech Republic; 3 Netherlands Cancer Institute and BOOG Study Center, IJsbaanpad 9, 1076 CV Amsterdam, The Netherlands; 4 University of Padova and Istituto Oncologico Veneto, IRCCS, Via Gattamelata, 64, Padua, Italy; 5 Vanderbilt-Ingram Cancer Center, 1301 Medical Center Dr #1710, Nashville, TN 37232 USA; 6 Edinburgh Cancer Centre, University of Edinburgh, Crewe Rd S, Edinburgh, EH4 2XR UK; 7 Sarah Cannon Research Institute, Fort Myers, FL 33916 USA; 8 University Hospital of Besançon, Jean-Minjoz University Hospital, 3 Boulevard Alexandre Fleming, 25000 Besançon, France; 9 Lillebaelt Hospital, Kabbeltoft 25, 7100 Vejle, Denmark; 10 Highlands Oncology Group, 3232 N Northhills Blvd, Fayetteville, AR 72703 USA; 11 Arizona Oncology, US Oncology Network, 3700 W State Rte 89A, Sedona, AZ 86336 USA; 12 Novartis Pharma AG, Fabrikstrasse 2, 4056 Basel, Switzerland; 13 Novartis Pharmaceuticals Corporation, 1 Health Plaza, East Hanover, NJ 07936 USA; 14 University of Texas MD Anderson Cancer Center, 1515 Holcombe Blvd, Houston, TX 77030 USA

**Keywords:** Breast cancer, CDK inhibitor, Ribociclib, Endocrine therapy, De novo advanced breast cancer, Hormone receptor-positive

## Abstract

**Purpose:**

Determine the efficacy and safety of first-line ribociclib plus letrozole in patients with de novo advanced breast cancer.

**Methods:**

Postmenopausal women with HR+ , HER2− advanced breast cancer and no prior systemic therapy for advanced disease were enrolled in the Phase III MONALEESA-2 trial (NCT01958021). Patients were randomized to ribociclib (600 mg/day; 3 weeks-on/1 week-off) plus letrozole (2.5 mg/day; continuous) or placebo plus letrozole until disease progression, unacceptable toxicity, death, or treatment discontinuation. The primary endpoint was investigator-assessed progression-free survival; predefined subgroup analysis evaluated progression-free survival in patients with de novo advanced breast cancer. Secondary endpoints included safety and overall response rate.

**Results:**

Six hundred and sixty-eight patients were enrolled, of whom 227 patients (34%; ribociclib plus letrozole vs placebo plus letrozole arm: *n* = 114 vs. *n* = 113) presented with de novo advanced breast cancer. Median progression-free survival was not reached in the ribociclib plus letrozole arm versus 16.4 months in the placebo plus letrozole arm in patients with de novo advanced breast cancer (hazard ratio 0.45, 95% confidence interval 0.27–0.75). The most common Grade 3/4 adverse events were neutropenia and leukopenia; incidence rates were similar to those observed in the full MONALEESA-2 population. Ribociclib dose interruptions and reductions in patients with de novo disease occurred at similar frequencies to the overall study population.

**Conclusions:**

Ribociclib plus letrozole improved progression-free survival vs placebo plus letrozole and was well tolerated in postmenopausal women with HR+, HER2− de novo advanced breast cancer.

## Introduction

Breast cancer diagnoses account for 25% of all newly diagnosed female cancer cases per year, affecting an estimated 1.7 million people worldwide [1]. The most common breast cancer subtype is hormone receptor-positive (HR+) disease, which constitutes 75% of all breast cancers [2]. Around 3–25% of newly diagnosed patients present with de novo HR+ advanced breast cancer [3, 4]. Patients are classed as having de novo advanced breast cancer if they present with advanced disease without having a previous diagnosis at an earlier stage of breast cancer; this excludes patients who have received prior therapy and relapsed. Current guidelines recommend the use of first-line endocrine therapy, with or without a cyclin-dependent kinase (CDK) 4/6 inhibitor, in patients with de novo or relapsed HR+ advanced breast cancer [5–8]. Due to a lack of exposure to systemic treatment, inherent differences may exist in the tumor biology of therapy-naïve de novo breast cancer compared with relapsed breast cancer [9], potentially contributing to the better prognosis of the de novo patient population [10]. The differences in clinical outcomes observed between patients with de novo and relapsed advanced breast cancer highlight the need to assess novel therapy options in this patient population [10–12].

The cyclin D–CDK4/6–inhibitor of CDK4 (INK4)–retinoblastoma (Rb) pathway is frequently disrupted in HR+ breast cancer [13], and has been associated with a poor clinical outcome and resistance to endocrine therapy [14]. Targeting the cyclin D–CDK4/6–INK4–Rb pathway may, therefore, present an effective strategy to enhance the efficacy of endocrine therapies. Ribociclib is an orally bioavailable, selective inhibitor of CDK4/6 [15]. Results from the Phase III MONALEESA-2 (NCT01958021) planned interim analysis demonstrated that the addition of ribociclib to letrozole significantly improved progression-free survival compared with placebo plus letrozole in patients with HR+, human epidermal growth factor receptor 2-negative (HER2−) advanced breast cancer, with a hazard ratio of 0.56 (*p* < 0.001) [16]. Treatment with ribociclib plus letrozole was associated with a rapid response; 76% of patients with evaluable measurable disease had a reduction in tumor size following 8 weeks of treatment [17]. Here we report efficacy and safety results from a prospective subgroup analysis of MONALEESA-2 in patients with de novo advanced breast cancer.

## Methods

### Study design and participants

MONALEESA-2 is a Phase III, randomized, double-blind, placebo-controlled trial that enrolled postmenopausal women with HR+, HER2− advanced breast cancer. Full details of the study design have been published previously [16]. Briefly, patients were required to have measurable disease with at least one measurable lesion as per Response Evaluation Criteria In Solid Tumors (RECIST) v1.1 [18] or at least one predominantly lytic bone lesion. All patients had an Eastern Cooperative Oncology Group (ECOG) performance status of 0 or 1. Patients were excluded if they had inflammatory breast cancer, central nervous system metastases, cardiac disease or Fridericia’s corrected QT interval (QTcF) > 450 ms, or impairment of gastrointestinal function that would have altered study drug absorption. Patients must not have received prior systemic therapy for advanced disease, except for ≤ 14 days of letrozole or anastrozole. The use of concomitant medications with known risk of prolonging the QT interval or inducing Torsades de Pointes was prohibited.

This study was conducted in accordance with Good Clinical Practice guidelines and the Declaration of Helsinki. An independent ethics committee and institutional review boards approved the study protocol and any subsequent amendments at each participating center. A study steering committee monitored study conduct in line with the protocol. Written informed consent was obtained from all the patients.

### Randomization

MONALEESA-2 patients from 223 centers in 29 countries were randomized 1:1 to receive oral ribociclib in combination with letrozole or placebo plus letrozole. Randomization was stratified according to the presence of liver and/or lung metastases. Screening and treatment allocation was performed using an interactive voice and web response system. Patients and investigators were blinded to the assigned treatment; both ribociclib and placebo were identical in label, packaging, appearance, and administration schedule. Treatment crossover from placebo to ribociclib was not permitted.

### Treatment and procedures

Patients received oral ribociclib (600 mg/day, 3 weeks-on/1 week-off schedule, in 28-day treatment cycles) in combination with letrozole (2.5 mg/day, continuously) or placebo plus letrozole until disease progression, unacceptable toxicity, death, or discontinuation for any other reason. Ribociclib dose adjustments including dose interruption, reduction, and permanent discontinuation were permitted for the management of adverse events. Dose modifications of letrozole were not permitted.

Tumor response was assessed locally according to RECIST v1.1 [18]. Computed tomography/magnetic resonance imaging assessments were conducted at screening, then every 8 weeks for the first 18 months, and every 12 weeks thereafter. A whole-body bone scan was performed at baseline. Cardiac function was monitored by triplicate electrocardiograms (ECGs) performed at screening, day 15 of cycle 1, and day 1 of cycles 2–3 in all patients; following a protocol amendment, additional ECG assessments were performed on day 1 of cycles 4–9 in all patients, and on day 1 of all subsequent cycles in patients who experienced a mean QTcF ≥ 481 ms prior to cycle 10. Centralized laboratory assessments including hematology and biochemistry panels were carried out at screening, cycle 1 day 15, and day 1 of all subsequent cycles; additional laboratory data were collected periodically throughout treatment. Adverse events were characterized throughout the study and graded according to the Common Terminology Criteria for Adverse Events (CTCAE) v4.03 [19].

### Outcomes

The primary objective was to compare progression-free survival between the two treatment arms per local investigator assessment. Overall survival was the key secondary endpoint. Additional secondary endpoints included overall response rate, clinical benefit rate, safety, and tolerability.

### Statistical analysis

This was a prespecified exploratory subgroup analysis. To compare the primary endpoint of progression-free survival between the treatment arms in the full study population, a log-rank test stratified according to the presence or absence of liver or lung metastases was utilized [16]. A stratified Cox regression analysis was used to estimate the hazard ratio and 95% confidence intervals of progression-free survival [16]. The subgroup analysis, including Kaplan–Meier estimates and treatment effect hazard ratio estimates using an unstratified Cox regression model, was performed in patients with de novo disease. Efficacy analyses were performed in the intent-to-treat population and safety analyses were performed in all patients who received at least one dose of study treatment and had at least one post-baseline safety assessment.

## Results

### Patient and disease characteristics

From January 24, 2014 to March 24, 2015, 668 patients were randomized to the ribociclib plus letrozole (*n* = 334) and placebo plus letrozole (*n* = 334) arms. Among all patients randomized, 227 (34%) presented with de novo advanced breast cancer at diagnosis; these patients were compared between treatment arms in this exploratory analysis. There was an even distribution of patients with de novo disease across both treatment arms; 114 (34%) patients in the ribociclib plus letrozole arm and 113 (34%) patients in the placebo plus letrozole arm (Table 1). Baseline characteristics of patients with de novo advanced breast cancer were well balanced across both treatment arms, except for ECOG performance status. A higher proportion of patients in the ribociclib plus letrozole arm (66%) had an ECOG performance status of 0, compared with 54% of patients in the placebo plus letrozole arm.

**Table 1:** Baseline patient characteristics

Characteristic	Patients with de novo advanced HR+, HER2− breast cancer *n* = 227	
	Ribociclib + letrozole (*n* = 114)	Placebo + letrozole (*n* = 113)
Age, median (range), years	62.5 (37.0–82.0)	63.0 (29.0–88.0)
Race, *n* (%)		
Caucasian	90 (79)	91 (81)
Asian	10 (9)	11 (10)
Black	6 (5)	4 (4)
Other/unknown	8 (7)	7 (6)
ECOG performance status, *n* (%)		
0	75 (66)	61 (54)
1	39 (34)	52 (46)
Disease stage at study entry, *n* (%)		
III	1 (1)	1 (1)
IV	113 (99)	112 (99)
Number of metastatic sites, *n* (%)		
0	1 (1)	0
1	33 (29)	36 (32)
2	37 (32)	38 (34)
≥ 3	43 (38)	39 (35)
Site of metastases, *n* (%)		
Breast	7 (6)	9 (8)
Bone	88 (77)	85 (75)
Bone only	28 (25)	24 (21)
Visceral^a^	53 (46)	55 (49)
Lymph nodes	61 (54)	56 (50)
Other	15 (13)	8 (7)

*ECOG* Eastern Cooperative Oncology Group, *HER2*− human epidermal growth factor receptor 2-negative, *HR*+ hormone receptor-positive

^a^Includes liver, lung, and other visceral metastases

### Treatment

**Fig. 1:**
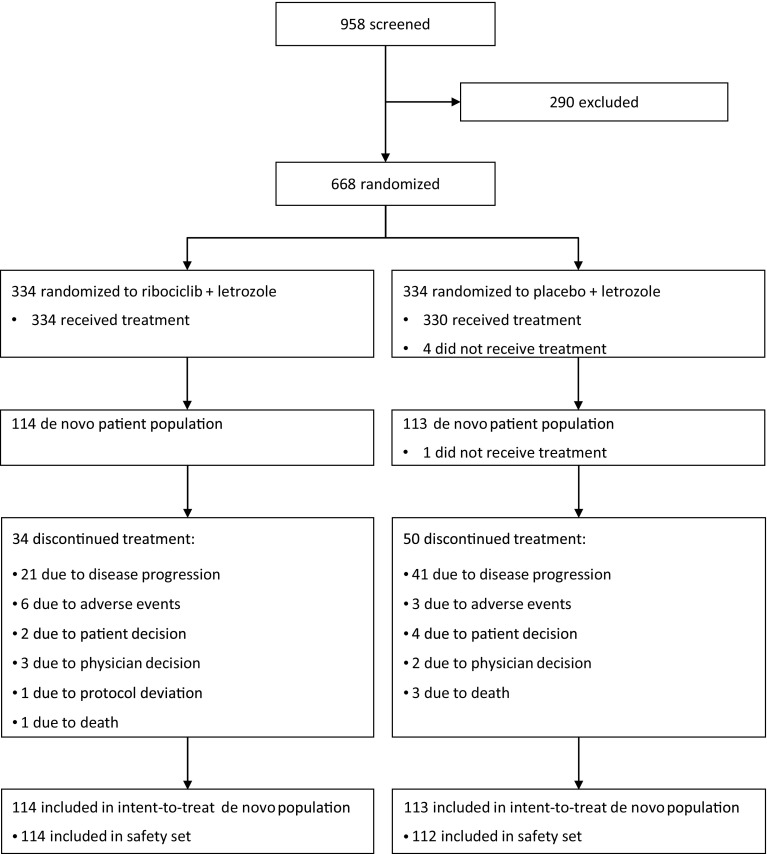
MONALEESA-2 trial profile (CONSORT diagram)

At the interim analysis data cut-off (January 29, 2016), fewer patients with de novo disease in the ribociclib plus letrozole arm had discontinued treatment compared with those receiving placebo plus letrozole (30% vs. 43%, respectively). The most common reason for treatment discontinuation was disease progression in both arms (Fig. 1). Patients in the ribociclib plus letrozole arm had a longer median duration of exposure to study treatment than patients who received placebo plus letrozole (14.1 and 12.8 months, respectively). The median relative dose intensity for ribociclib was 88% and the median relative dose intensity for letrozole was 100% for both treatment arms.

### Efficacy

**Fig. 2:**
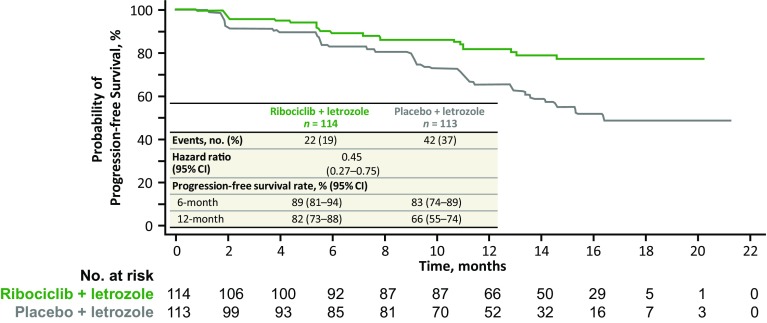
Kaplan–Meier analysis of locally assessed progression-free survival in patients with de novo advanced breast cancer in the MONALEESA-2 trial. *CI* confidence interval

The combination of ribociclib plus letrozole prolonged progression-free survival compared with placebo plus letrozole in patients with de novo advanced breast cancer, with a hazard ratio of 0.45 (95% CI 0.27–0.75). Median progression-free survival in the ribociclib plus letrozole arm was not reached versus 16.4 months in the placebo plus letrozole arm (Fig. 2). After 12 months, the estimated progression-free survival rates in the ribociclib plus letrozole versus placebo plus letrozole arms were 82% versus 66%, respectively. The overall response rates in the intent-to-treat population were 47% in the ribociclib plus letrozole arm versus 34% in the placebo plus letrozole arm; clinical benefit rates were 83% versus 77% (Table 2). In patients with measurable disease at baseline, the overall response rates were 56% versus 45% and the clinical benefit rates were 82% versus 77% in the ribociclib plus letrozole versus placebo plus letrozole arms, respectively. Overall survival results were immature at the time of this interim analysis.

**Table 2:** Summary of best overall response per RECIST v1.1

	Patients with de novo advanced HR+, HER2− breast cancer *n* = 227	
	Ribociclib + letrozole	Placebo + letrozole
All patients, *n*	114	113
Confirmed BOR, *n* (%)		
CR	2 (2)	1 (1)
PR	52 (46)	37 (33)
SD	36 (32)	34 (30)
NCRNPD	16 (14)	26 (23)
PD	3 (3)	9 (8)
Unknown	5 (4)	6 (5)
ORR^a^, *n* (%) [95% CI]	54 (47) [38–57]	38 (34) [25–42]
CBR^b^, *n* (%) [95% CI]	95 (83) [77–90]	87 (77) [69–85]
Patients with measurable disease at baseline, *n*	96	83
Confirmed BOR, *n* (%)		
CR	2 (2)	0
PR	52 (54)	37 (45)
SD	36 (38)	34 (41)
PD	2 (2)	8 (10)
Unknown	4 (4)	4 (5)
ORR^a^, *n* (%) [95% CI]	54 (56) [46–66]	37 (45) [34–55]
CBR^b^, *n* (%) [95% CI]	79 (82) [75–90]	64 (77) [68–86]

*BOR* best overall response, *CBR* clinical benefit rate, *CI* confidence interval, *CR* complete response, *HER2*− human epidermal growth factor receptor 2-negative, *HR*+ hormone receptor-positive, *NCRNPD* neither complete response nor progressive disease (for non-measurable disease at baseline), *ORR* overall response rate, *PD* progressive disease, *PR* partial response, *SD* stable disease

^a^CR + PR

^b^CR + PR + (SD + NCRNPD ≥ 24 weeks)

### Safety and tolerability

Overall, the safety profile of ribociclib plus letrozole in patients with de novo advanced breast cancer was similar to that observed in the full MONALEESA-2 study population [16]. In the de novo subset, there was a similar incidence of all-grade adverse events of any causality in the ribociclib plus letrozole vs placebo plus letrozole treatment arms (97% vs. 97%). Neutropenia, nausea, and fatigue were the most common adverse events in the ribociclib plus letrozole arm (Table 3). The incidence of non-hematologic adverse events of nausea, fatigue, alopecia, vomiting, rash, pyrexia, elevated aspartate aminotransferase, and elevated alanine aminotransferase was increased by > 10% in the ribociclib plus letrozole arm compared with the placebo plus letrozole arm.

**Table 3:** Adverse events (any grade; ≥ 15% in either arm), regardless of relationship to study drug

*n* (%)	Patients with de novo advanced HR+, HER2− breast cancer *n* = 226			
	Ribociclib + letrozole (*n* = 114)		Placebo + letrozole (*n* = 112^a^)	
Grade	All grade	Grade 3/4	All grade	Grade 3/4
Any adverse event	111 (97)	88 (77)	109 (97)	35 (31)
Neutropenia^b^	80 (70)	63 (55)	5 (4)	1 (1)
Nausea	55 (48)	1 (1)	29 (26)	0
Fatigue	48 (42)	1 (1)	30 (27)	1 (1)
Alopecia	45 (39)	–	17 (15)	–
Leukopenia^c^	36 (32)	24 (21)	0	0
Diarrhea	32 (28)	2 (2)	24 (21)	0
Vomiting	29 (25)	2 (2)	17 (15)	0
Anemia^d^	28 (25)	2 (2)	4 (4)	3 (3)
Rash^e^	27 (24)	2 (2)	11 (10)	0
Arthralgia	25 (22)	0	37 (33)	0
Back pain	25 (22)	1 (1)	22 (20)	0
Headache	25 (22)	1 (1)	24 (21)	0
Constipation	24 (21)	1 (1)	18 (16)	0
Hot flush	24 (21)	0	27 (24)	0
Decreased appetite	22 (19)	2 (2)	21 (19)	1 (1)
Hypertension	20 (18)	15 (13)	16 (14)	13 (12)
Pyrexia	20 (18)	1 (1)	6 (5)	0
AST increased	19 (17)	7 (6)	4 (4)	0
Cough	17 (15)	0	19 (17)	0

*AST* aspartate aminotransferase, *HER2*− human epidermal growth factor receptor 2-negative, *HR*+ hormone receptor-positive

^a^One patient in the placebo plus letrozole arm was randomized but did not receive study treatment

^b^Includes neutropenia, neutrophil count decreased, and granulocytopenia

^c^Includes leukopenia and white blood cell count decreased

^d^Includes anemia, hemoglobin decreased, and anemia macrocytic

^e^Includes rash and rash maculopapular

There was a higher incidence of Grade 3/4 adverse events in the ribociclib plus letrozole arm (77%) compared with the placebo plus letrozole arm (31%). The most common Grade 3/4 adverse events (≥ 15%) in the ribociclib plus letrozole arm were neutropenia (55%) and leukopenia (21%). Febrile neutropenia rates were low, occurring in 2 (2%) versus 0 patients with de novo disease treated with ribociclib plus letrozole vs placebo plus letrozole. QTcF prolongation > 500 ms was not reported in any patients with de novo disease in either treatment arm. Adverse events were the most common reason for dose interruptions and reductions. At least one dose interruption due to an adverse event occurred in 75 (66%) patients in the ribociclib plus letrozole arm and 17 (15%) patients in the placebo plus letrozole arm. At least one dose reduction due to an adverse event occurred in 55 (48%) patients in the ribociclib plus letrozole arm and 6 (5%) patients in the placebo plus letrozole arm. Neutropenia was the most frequent adverse event requiring ribociclib dose interruption or dose reduction (56 [49%] patients).

## Discussion

This predefined subgroup analysis of the MONALEESA-2 trial demonstrates that postmenopausal women with de novo HR+, HER2− advanced breast cancer at diagnosis who received ribociclib in combination with letrozole had prolonged progression-free survival compared with those who received placebo plus letrozole, with an approximate 55% reduction in the risk of progression (hazard ratio 0.45, 95% CI 0.27–0.75). Median progression-free survival was not reached in the ribociclib plus letrozole arm, compared with 16.4 months in the placebo plus letrozole arm. Progression-free survival benefit with the addition of ribociclib to letrozole was consistent with that observed in the MONALEESA-2 intent-to-treat population, where a hazard ratio of 0.56 (95% CI 0.43–0.72, *p* < 0.001) was observed [16]. Ribociclib plus letrozole was also associated with improved overall response and clinical benefit rates versus placebo plus letrozole in patients with de novo disease.

Enhanced treatment benefits in patients with de novo advanced disease have previously been described with the CDK4/6 inhibitor palbociclib in combination with letrozole in the PALOMA-2 trial [20]. The FALCON study recently reported improved efficacy for first-line fulvestrant over anastrozole, with a hazard ratio of 0.80 (95% CI 0.64–0.99) in a largely de novo patient population [21]. Median progression-free survival was 16.6 months in patients who received fulvestrant therapy compared with 13.8 months in the anastrozole arm; both values are in line with the median progression-free survival of 16.4 months reported herein for placebo plus letrozole-treated patients with de novo advanced disease.

CDK4/6 inhibitor combinations have demonstrated significantly extended progression-free survival in patients who received no prior systemic therapy for advanced disease in the MONALEESA-2 [16] and PALOMA-2 [22] trials, highlighting the utility of this treatment strategy in the first-line setting for advanced HR+, HER2− breast cancer. Recent data have also shown promising results for CDK4/6 inhibitor-based regimens in patients with HR+, HER2− breast cancer who received prior single-agent endocrine therapy. Abemaciclib in combination with fulvestrant extended progression-free survival compared with fulvestrant monotherapy, [23] and palbociclib plus fulvestrant also improved progression-free survival compared with fulvestrant plus placebo [24]. Data from these studies demonstrate the potential for CDK4/6 inhibitor-based combinations as second-line therapies for patients with advanced HR+, HER2− breast cancer who have received prior single-agent endocrine therapy. Further research is required to determine the optimal treatment sequence for CDK4/6 inhibitors in advanced HR+, HER2− breast cancer, particularly since current data in the second-line setting are from patients who have received no prior CDK4/6 inhibitor-based therapy. Final overall survival analyses are needed to determine whether a potential long-term benefit is observed with the addition of a CDK4/6 inhibitor to first-line endocrine therapy, and results are eagerly awaited.

The adverse events associated with the combination of ribociclib plus letrozole in patients with de novo disease were consistent with those observed in the overall MONALEESA-2 study population [16] and the safety profile of other CDK4/6 inhibitors [22, 24]. The hematologic toxicities of neutropenia, leukopenia, and anemia were among the most frequent adverse events in the ribociclib plus letrozole arm, consistent with the known on-target effect of CDK4/6 inhibitors on hematologic precursors in the bone marrow [25]. Grade 3/4 neutropenia was common in patients with de novo disease-receiving ribociclib; however, the condition was rapidly reversible upon dose reduction or interruption.

In conclusion, combining ribociclib with letrozole provided clinically meaningful improvements in progression-free survival, overall response rates, and clinical benefit rates and was well tolerated in patients with de novo advanced HR+, HER2− breast cancer. Results from this subgroup analysis of the MONALEESA-2 trial demonstrate that ribociclib plus letrozole provides a valuable first-line therapy option for women who present with de novo HR+, HER2− advanced breast cancer at diagnosis.
